# Genome methylation and regulatory functions for hypoxic adaptation in Tibetan chicken embryos

**DOI:** 10.7717/peerj.3891

**Published:** 2017-10-06

**Authors:** Yawen Zhang, Wenyu Gou, Jun Ma, Hongliang Zhang, Ying Zhang, Hao Zhang

**Affiliations:** National Engineering Laboratory for Animal Breeding/Beijing Key Laboratory for Animal Genetic Improvement, China Agricultural University, Beijing, China

**Keywords:** Tibetan chicken, Hypoxia adaption, MeDIP-seq, DNA methylation, Regulatory genes

## Abstract

Tibetan chickens have unique adaptations to the extreme high-altitude environment that they inhabit. Epigenetic DNA methylation affects many biological processes, including hypoxic adaptation; however, the regulatory genes for DNA methylation in hypoxic adaptation remain unknown. In this study, methylated DNA immunoprecipitation with high-throughput sequencing (MeDIP-seq) was used to provide an atlas of the DNA methylomes of the heart tissue of hypoxic highland Tibetan and lowland Chahua chicken embryos. A total of 31.2 gigabases of sequence data were generated from six MeDIP-seq libraries. We identified 1,049 differentially methylated regions (DMRs) and 695 related differentially methylated genes (DMGs) between the two chicken breeds. The DMGs are involved in vascular smooth muscle contraction, VEGF signaling pathway, calcium signaling pathway, and other hypoxia-related pathways. Five candidate genes that had low methylation (*EDNRA*, *EDNRB2*,* BMPR1B*,* BMPRII*, and *ITGA2*) might play key regulatory roles in the adaptation to hypoxia in Tibetan chicken embryos. Our study provides significant explanations for the functions of genes and their epigenetic regulation for hypoxic adaptation in Tibetan chickens.

## Introduction

The molecular mechanisms underlying hypoxic adaptation in highland animals have long garnered attention for biological and medical research, not only because of the evolutionary significance of high-altitude adaptation but also in the context of understanding hypoxia in relation to human diseases. Adaptation to hypoxia in animals is a complex trait that involves multigenes and multi-channel regulatory mechanisms. Many indigenous animals, such as the Tibetan antelope (Ge et al., 2013), ground tit (Cai et al., 2013; Qu et al., 2013), yak (Qiu et al., 2012), pig (Li et al., 2013), dog (Gou et al., 2014; Wang et al., 2014), snub-nosed monkey (Yu et al., 2016), and fish (Wang et al., 2015b), have been studied to identify selective signatures and functional genes for adaptation to high altitudes. The Tibetan chicken (*Gallus gallus*), a unique breed native to the Qinghai–Tibet Plateau, shows distinctive genetic adaptation to the high-altitude environment in which it is found. The increased hatchability of the Tibetan chicken compared with lowland breeds under hypoxia, even for individuals that have migrated to and remained at lower altitudes for several years, shows the stable genetic mechanism underlying hypoxic adaptation (Zhang et al., 2008a). Tibetan chicken embryos have the physical foundations for hypoxic adaptation, including hatchability, mitochondrial respiratory function, embryonic organ system development, and oxidoreductase (Bao et al., 2007; Jia et al., 2016; Zhang & Burggren, 2012; Zhang et al., 2008a). Furthermore, genome variation analysis has identified a series of pathways and genes related to hypoxia in the Tibetan chicken (Wang et al., 2015a; Zhang et al., 2016). However, the regulatory mechanisms of these hypoxic adaptive genes remain unclear.

DNA methylation is a central epigenetic modification that plays an essential regulatory role in cellular processes (Lister et al., 2009). In mammals, DNA methylation occurs predominantly at the 5′ position of cytosines in CpG dinucleotides, which are sparsely distributed throughout the genome, except in short genomic regions called CpG islands (CGIs) (Mason et al., 2012). The state of CpG methylation stabilizes chromatin structure, and possibly inhibits the accessibility of these DNA regions to transcription factors and regulates related gene expression (Clark & Melki, 2002). DNA methylation plays an important role in hypoxia-inducible factors (HIFs), such as HIF-α stabilization and HIF-1 binding, as well as in regulating the transcription of hypoxia response genes (Watson et al., 2010). A previous study showed significant genome-wide epigenetic differences between Ethiopians living at high and low altitudes (Alkorta-Aranburu et al., 2012). CpG methylation of the estrogen receptor-α promoter is increased under chronic hypoxia in the uterine arteries of pregnant sheep, inhibiting gene expression (Dasgupta et al., 2012). Therefore, we propose that DNA methylation participates in the regulation and expression of hypoxia-adaptive genes.

The heart is the key organ that stimulates growth under hypoxia during chicken embryonic development (Zhang & Burggren, 2012). In this study, we use methylated DNA immunoprecipitation and high-throughput sequencing (MeDIP-seq) to generate an atlas of DNA methylomes for the heart tissue of hypoxic chicken embryos to investigate the difference in DNA methylation between TC and their lowland counterparts, Chahua chickens (CH), which show extreme differences in hypoxic adaptation compared with TC. The objective of the present study was to identify differentially methylated regions (DMRs) and related genes that might be involved in regulatory functions for hypoxic adaptation in TC.

## Materials and Methods

### Sample collection

Eggs of TC and CH were collected from the Experimental Chicken Farm of the China Agricultural University (CAU). The eggs (150 for each group) were placed in a hypoxic (13% O_2_) incubator, with temperature and humidity of 37.8 °C and 60%, respectively. Embryonic heart tissue was collected after 16 days under incubation and stored at −80 °C. All experimental procedures and sample collections were approved by the State Key Laboratory for Agro-biotechnology of CAU (Permit Number: XK257).

### Methylated DNA immunoprecipitation and sequencing

Genomic DNA was extracted from the heart tissue using the phenol–chloroform method and sonicated to obtain fragments in the range of 100–500 bp. The double-stranded DNA was denatured and subsequently immunoprecipitated with an antibody that specifically recognizes 5′-methylcytosine using a Magnetic Methylated DNA Immunoprecipitation Kit (Diagenode, Liege, Belgium). The specificity of the enrichment was confirmed by quantitative real-time PCR (qRT-PCR). After PCR amplification of the enriched fragments, we selected 220–320 bp fragments and quantified these using an Agilent 2100 Analyzer (Agilent Technologies, Palo Alto, CA, USA). Sequencing libraries of MeDIP fragments were constructed by adopting the Illumina paired-end protocol, consisting of end repair, <A>  base addition, and adaptor ligation steps, performed using Illumina’s Paired-End DNA Sample Prep kit according to the manufacturer’s instructions. The MeDIP-seq library was subjected to paired-end sequencing using a HiSeq 2500 sequencer (Illumina, San Diego, CA, USA) at a 125 bp read length. Three individual female embryo libraries each were prepared as biological replicates for TC and CH.

### Genome mapping of MeDIP-seq reads

Raw sequencing data were filtered by removing the adaptors and discarding low-quality reads that contained more than 10% of undetermined bases (N) or over 40% of low-quality bases (Qphred ⩽ 20) (A Gordon & G Hannon, 2010, unpublished data). The clean reads were aligned to the chicken reference genome (version 4.74; ftp://ftp.ensembl.org/pub/release-74/fasta/gallus_gallus/dna/) using SOAP2 (version 2.21) with no more than five mismatched bases (Li et al., 2009). Uniquely mapped reads were used for the following analyses. The MeDIP-Seq data from this study have been submitted to the NCBI Gene Expression Omnibus under accession no. GSE99129.

### Distribution analysis of MeDIP-seq reads

According to GTF annotation version 4.74 (ftp://ftp.ensembl.org/pub/release-74/gtf/gallus_gallus/Gallus_gallus.Galgal4.74.gtf.gz), the reads were distributed in chromosomes or in components of the genome, including the upstream 2 kb region, 5′-untranslated regions (UTRs), coding DNA sequences (CDSs), introns, 3′-UTRs, and the downstream 2 kb region. The region from the transcript start site (TSS) to the transcript termination site (TTS) was defined as the gene body region. The regions 2 kb upstream of the TSS and 2 kb downstream of the TTS were split into 20 non-overlapping equal windows, whereas the gene bodies were split into 40 equal windows. The regions both 2 kb upstream and downstream of CGIs were split into 20 non-overlapping equal windows, and the CGIs were split into 40 equal windows. The average alignment depth was calculated for each window and was normalized by dividing by 1,000. The CGIs were scanned by CpGPlot (http://www.ebi.ac.uk/Tools/seqstats/emboss_cpgplot/) (Goujon et al., 2010) using standard settings (length > 200 bp, G  + C > 50%, and CpG_O∕E_ > 0.6) (Gardinergarden & Frommer, 1987).

### Peak calling and identification of DMRs

Model-based Analysis of ChIP-Seq (MACS1.4.0) was used to scan the enriched peaks where the reads were mapped to the same position in the genome (Feng et al., 2012; Zhang et al., 2008b). The bam files of three biological replicates were combined into one sample, and then the bam files of each TC and CH were used to call peaks with MACS, respectively. The TC and CH peaks were merged as total methylated regions. If the peaks detected in the two groups did not overlap or overlapped by less than 50% of the shorter peak length, the peaks were classified as unique peaks. If the peaks of the two groups overlapped by more than 50% of the shorter peak length, the peaks were merged into one peak. Numbers of reads within a peak were compared between TC and CH using a chi-square test, in which *p* values were corrected by a false discovery rate to avoid false positives. The FC (fold change) is the ratio of the numbers of read counts in the peak of TC to that of CH. DMRs were identified using a filter standard of *p* < 0.01, false discovery rate < 0.01, and |log_2_FC|⩾1.

### Functional annotation of DMRs

We annotated differentially methylated genes (DMGs) on the basis of at least one DMR located in the gene body, the region 2 kb upstream, and the region 2 kb downstream, using downloaded annotation data from the University of California Santa Cruz (UCSC) Genome Browser Database (http://genome.ucsc.edu). The human orthologous gene ensemble IDs of these DMGs were submitted to the Database for Annotation, Visualization, and Integrated Discovery (DAVID) web server (http://david.abcc.ncifcrf.gov/) to perform functional enrichment analysis with gene ontology (GO) and Kyoto Encyclopedia of Genes and Genomes (KEGG) pathway categories (Dennis et al., 2003).

### Validation of DMG expression using qRT-PCR

To verify the regulatory relationship between DNA methylation and gene expression, the expression of several DMGs was measured by qRT-PCR using eight replicates for each of the TC and CH groups. Total RNA was isolated from the heart tissue using TRIzol (Invitrogen, Carlsbad, CA, USA) and reverse transcribed to cDNA using a FastQuant RT Kit (with gDNase; Tiangen, Beijing, China). Five pairs of primers for qRT-PCR were designed using Primer Premier 5.0 software (Table S1), including HPRT, which served as the reference gene. Gene expression levels of the five genes were calculated using the 2^−ΔΔCt^ method. Differences in the expression of each gene between TC and CH were calculated and compared with the FC of gene methylation in MeDIP-seq.

## Results

### Summary of MeDIP-seq data

We generated 31.2 gigabases (Gb) of clean MeDIP-seq data from six samples (approximately 5.2 Gb per sample), and approximately 93.79% (92.66%–94.91%) of the data had Qphred quality scores of >20 (Table S2). Approximately 66.6% of the clean reads were aligned on the chicken genome, and 57.1% of the clean reads were uniquely mapped (Table S3). With the exception of some gaps, the MeDIP-seq reads were distributed across most chromosomal regions (chromosomes 1–28, 32, MT, W, and Z) in each sample (Fig. S1). The distribution density of the MeDIP-Seq reads in the 1 kb windows was the highest in those containing 5–10 CpGs across all samples (Fig. S2). The distribution of MeDIP-seq reads in the different genome elements (upstream 2 kb, 5′-UTR, CDS, intron, 3′-UTR, and downstream 2 kb) showed that the reads were distributed among all the genomic feature regions (Fig. S3).

**Figure 1:**
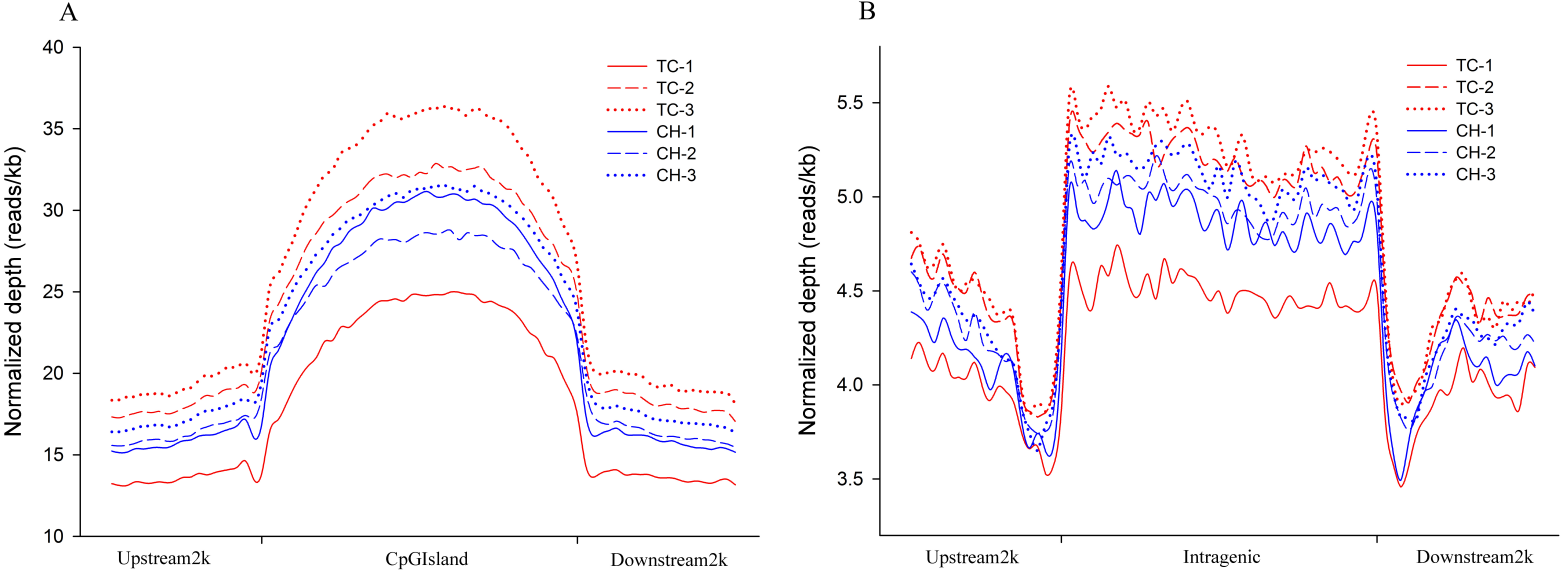
Distribution of MeDIP-Seq reads in different genomic regions. (A) Distribution of MeDIP-Seq reads in CpG islands and the regions 2 kb upstream and downstream in Tibetan chicken (TC) and Chahua chicken (CH). The *x*-axis indicates the position of reads on CpG islands, and the *y*-axis indicates the normalized read number. (B) Distribution of MeDIP-Seq reads in the intragenic region and the regions 2 kb upstream and downstream in Tibetan chicken (TC) and Chahua chicken (CH). The *x*-axis indicates the position around gene bodies, and the *y*-axis indicates the normalized read number.

The distribution of the MeDIP-seq reads in the CGI and its adjacent regions showed a higher level of DNA methylation than the regions 2 kb upstream and downstream of the CGIs (Fig. 1A). The distribution of MeDIP-seq reads in the region 2 kb upstream from the TSS, the intragenic region (from TSS to TTS), and the region 2 kb downstream from the TTS showed that the intragenic region displayed a higher level of DNA methylation than the 5′- and 3′-flanking regions of genes. Two valleys of DNA methylation were observed in the upstream region adjacent to the TSS and the downstream region adjacent to the TTS, respectively (Fig. 1B). The observed DNA methylation pattern is in accordance with that observed in previous studies (Huang et al., 2014; Lorincz et al., 2004; Rountree & Selker, 1997; Zilberman et al., 2007), indicating that this is a common mechanism for the regulation of gene expression that has been conserved among species.

### Methylated peaks in the genomes of TC and CH

By scanning peaks on a genome-wide scale, we obtained 103,293 peaks in TC and 107,920 peaks in CH, i.e., the total number of peaks was lower in TC, and the peaks covered 13.78% and 15.25% of the genome, respectively (Table S4).

### Differentially methylated regions

A total of 1,049 DMRs were detected between TC and CH, including 381 up-methylated and 668 down-methylated DMRs in TC. Furthermore, across all the DMRs, 94 were in the promoter (the region 2 kb upstream from the TSS), including 41 down-methylated and 53 up-methylated DMRs in TC.

**Figure 2:**
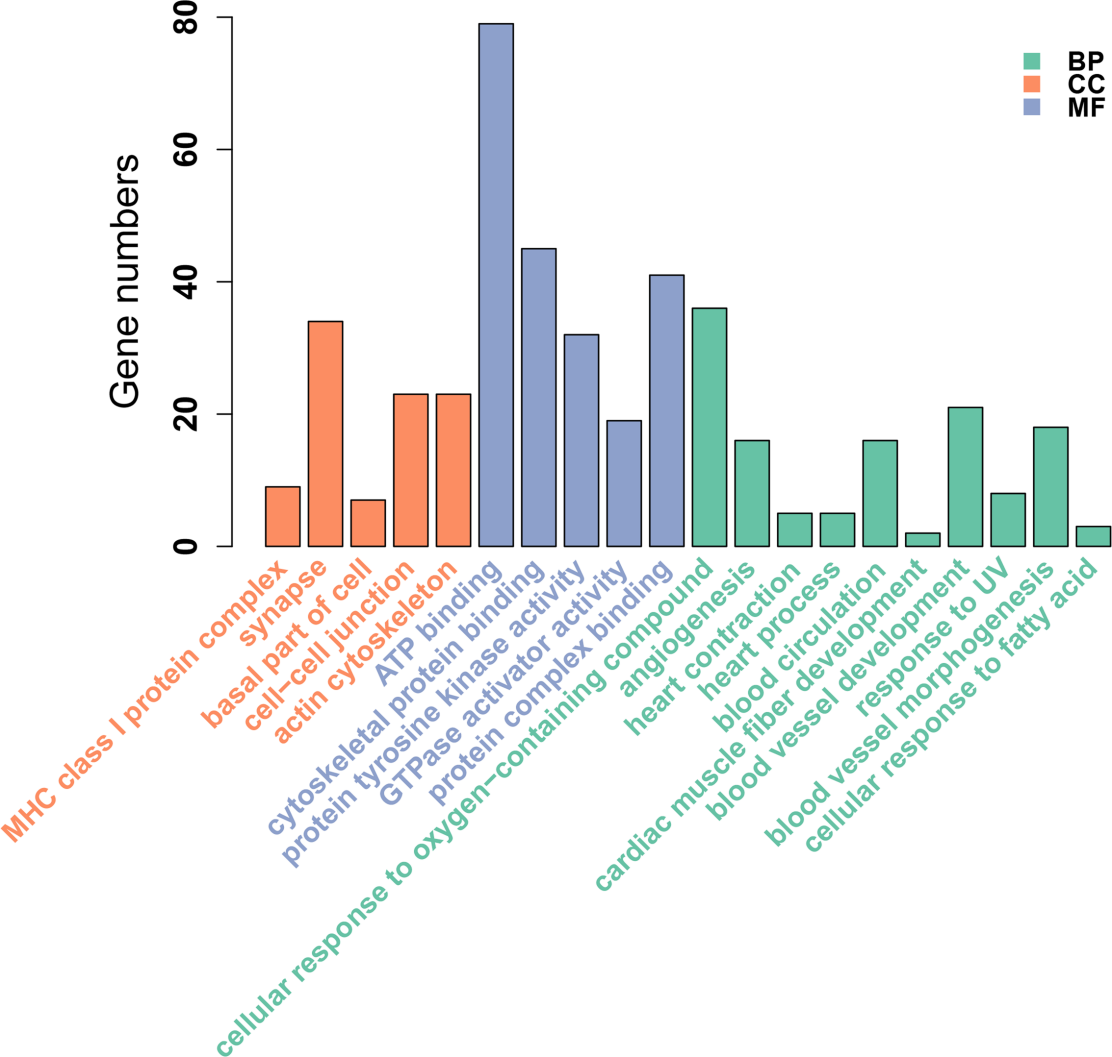
GO enrichment analysis of DMGs between Tibetan chicken (TC) and Chahua chicken (CH). The *x*-axis shows the GO enrichment terms, and the *y*-axis represents the gene numbers. The green bars represent biological processes (BP); the orange bars represent the cellular component (CC); and the blue bars represent the molecular functions (MF) of the GO terms.

### Functional annotation of the DMRs

**Figure 3:**
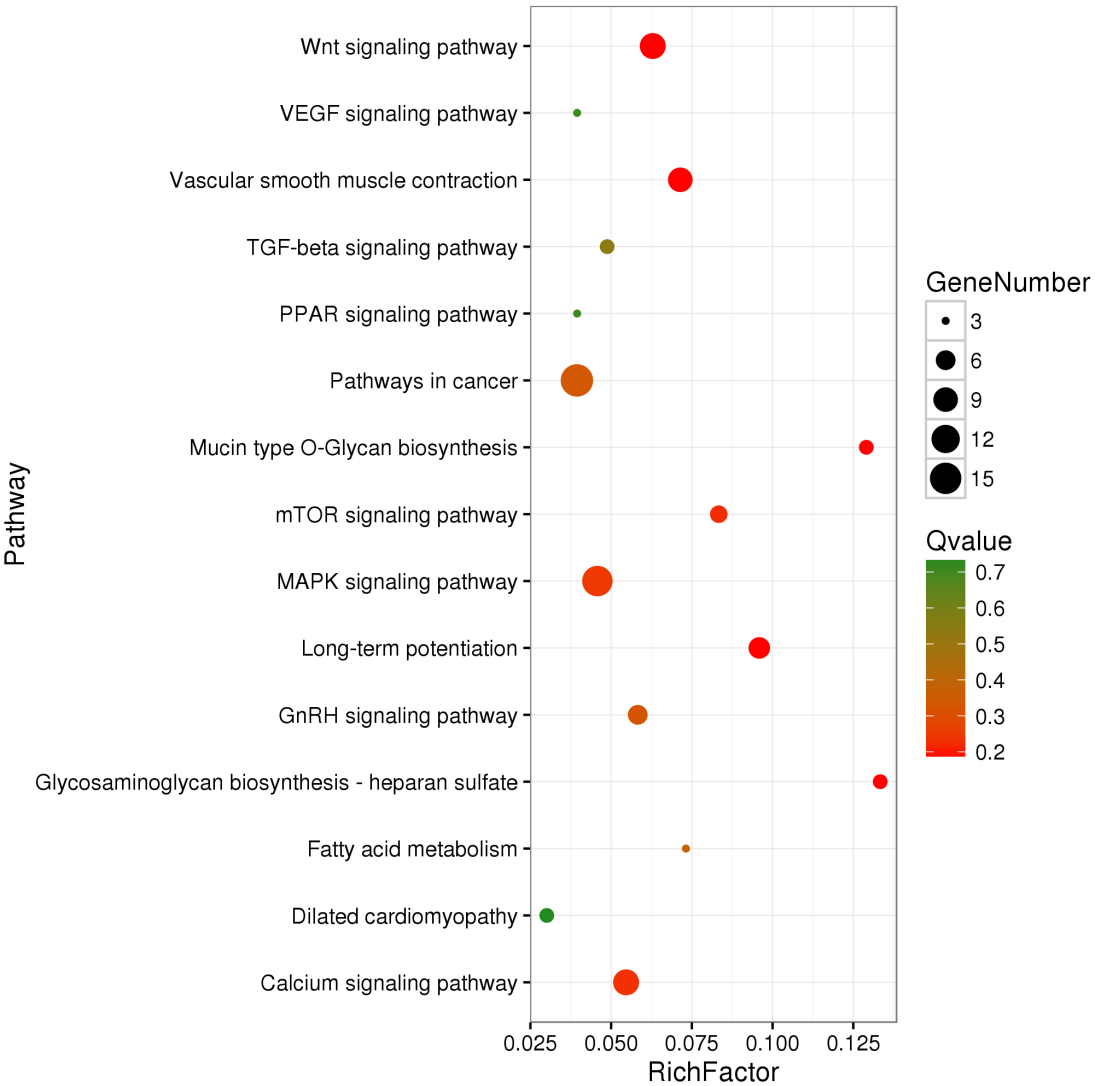
KEGG-enriched scatter plot of DMGs between Tibetan chicken (TC) and Chahua chicken (CH). The *x*-axis represents the rich factor. The rich factor is the ratio of differentially methylated gene (DMG) numbers annotated in this pathway term to the total gene numbers annotated in this pathway term. The *y*-axis shows the pathway enrichment terms. The *Q*-value represents the corrected *p* value, where a smaller *Q*-value indicates a higher significance.

We identified 695 DMGs between TH and CH (Table S5) based on the annotation of the 1,049 DMRs. The enriched GO terms of the 695 DMGs included vascular development, heart contraction, energy metabolism, cell adhesion, and inflammation, amongst other biological processes (Fig. 2, Table S6). In addition, KEGG pathway analysis showed that there were 168 enriched pathways (Table S7), which mainly involved vascular smooth muscle contraction, VEGF signaling pathway, calcium signaling pathway, TGF-beta signaling pathway, fatty acid metabolism, and other upstream and downstream signaling pathways of HIF signaling (Fig. 3). Taking gene functional annotation into consideration, five key DMGs were identified (*BMPR1B*, *BMPRII*, *ITGA2*, *EDNRA*, and *ENDRB2*), which might play important roles in adaptation to hypoxia in TC. Two genes (*BMPR1B* and *BMPRII*) are associated with ATP binding and the TGF-beta signaling pathway. Two other DMGs (*ENDRA* and *ENDRB2*) are associated with calcium signaling pathway, with *EDNRA* also playing a role in vascular smooth muscle contraction. *ITGA2* is involved in pathways in cancer and dilated cardiomyopathy (Table 1).

### Validation of the expression of the DMGs

**Figure 4:**
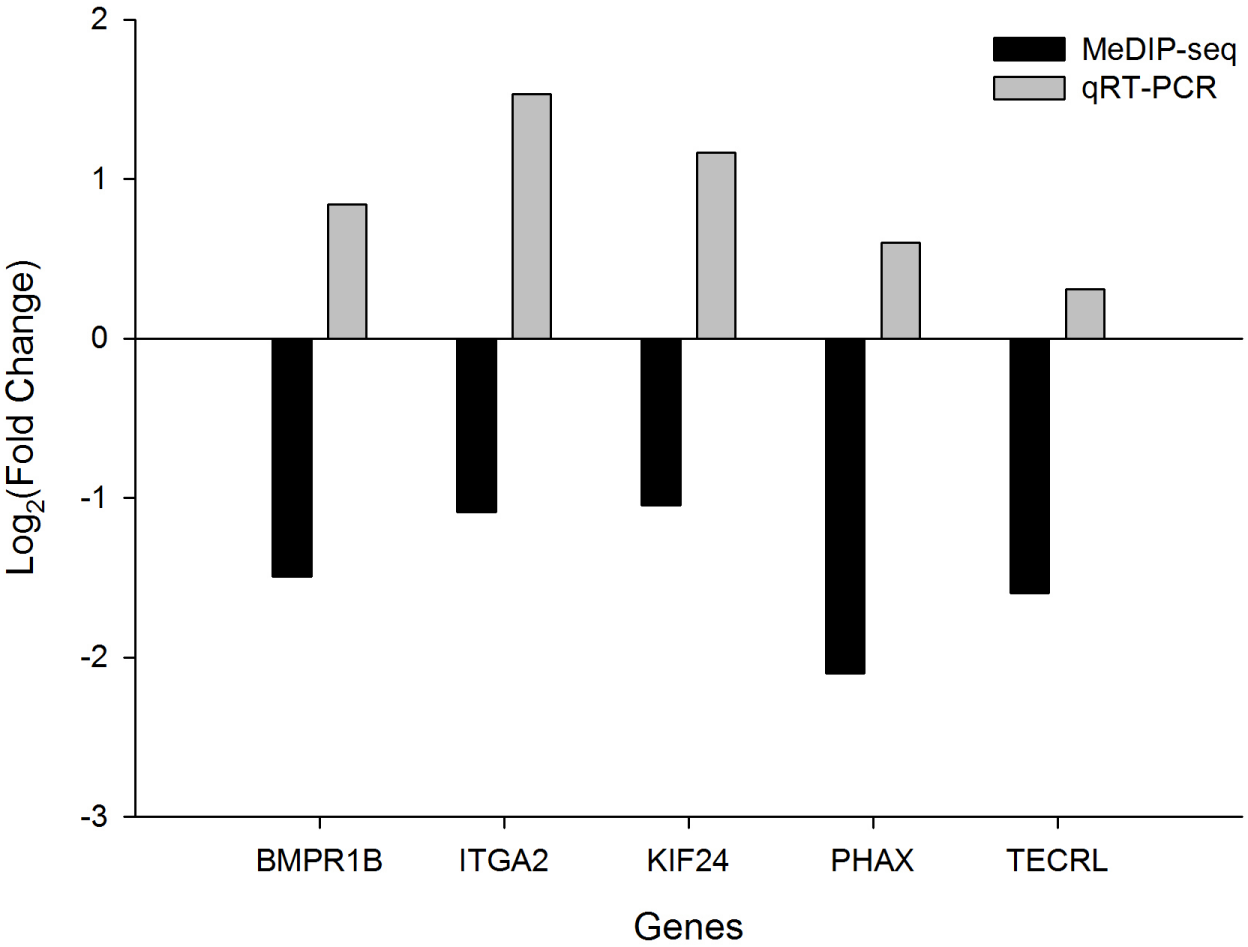
The fold change (FC) in mRNA expression level of five differentially methylated genes (DMGs) in Tibetan chicken (TC) and Chahua chicken (CH). The *x*-axis represents the DMGs, and the *y*-axis represents the log_2_(FC) of gene expression level and the log_2_(FC) of the DNA methylation between TC and CH.

We selected five DMGs to detect the gene expression levels in the heart tissue of chicken embryos by qRT-PCR: bone morphogenetic protein receptor type 1B (*BMPR1B*), integrin subunit alpha 2 (*ITGA2*), kinesin family member 24 (*KIF24*), phosphorylated adaptor for RNA export (*PHAX*), and trans-2,3-enoyl-CoA reductase-like (*TECRL*). All these genes showed downregulated methylation and had higher expression levels in TC compared with CH (Fig. 4). In particular, the expression levels of *BMPR1B*, *ITGA2*, and *KIF24* differed significantly between TC and CH (Fig. S4), suggesting that the DMGs identified in MeDIP-seq were reliable, and that downregulated DNA methylation leads to the upregulation of gene expression.

**Table 1:** Potential key differentially methylated genes (DMGs) and their functions related to hypoxic adaption in the Tibetan chicken.

Gene	Log_2_FC (TC/CH)	FDR	Differential methylated region	Functional analysis
*BMPR1B*	−1.493	4.80E−55	Five-UTR	ATP binding; TGF-beta signaling pathway
*BMPRII*	−1.082	2.45E−24	Intron	ATP binding; TGF-beta signaling pathway
*ITGA2*	−1.091	1.36E−22	Upstream2k	Pathways in cancer; dilated cardiomyopathy
*EDNRA*	−1.166	1.44E−25	Downstream2k	Angiogenesis; blood circulation; blood vessel development; blood vessel morphogenesis; calcium signaling pathway; vascular smooth muscle contraction
*EDNRB2*	−2.478	4.83E−80	CDS	Calcium signaling pathway

**Notes.**

TC Tibetan chicken CH Chahua chicken

## Discussion

Over the past two decades, HIF signaling and its cascading pathways have been recognized as playing key roles in high-altitude adaptation (Simonson et al., 2010; Storz, 2010). HIF-dependent physiological adaptations assist systemic oxygen homeostasis under hypoxic conditions (Majmundar, Wong & Simon, 2010). Some of the DMGs we detected in the present study are involved in biological processes that are mediated by HIFs, including cardiovascular development, vascular smooth muscle contraction, heart contraction, blood vessel morphogenesis, angiogenesis, and the VEGF signaling pathway. Endothelin 1 (*EDN1*) is one of the most effective vasoconstrictors (Vadapalli et al., 2010). In mammals, two independent endothelin receptors, *EDNRA* and *EDNRB* (Thaete et al., 2007), have been linked to high-altitude pulmonary edemas (Sharma, Singh & Sarkar, 2014), as well as hypertension, cardiac hypertrophy, and coronary artery disease (Schneider, Boesen & Pollock, 2007). *ENDRA*, which is associated with HIF activity, is a positively selected gene in Tibetan (Simonson et al., 2010). In the present study, the Tibetan chicken embryos had relatively low methylated loci in *DENRA* and *DENRB2* genes under hypoxic conditions, which might up-regulate the gene expression levels and maintain heart development and functions. Therefore, we suggest these the two genes, modified by DNA methylation, participate in the regulation of cardiovascular development in Tibetan chicken embryos under hypoxic conditions.

These endothelin receptor genes are also detected in the calcium signaling pathway, and the calcium signaling pathway was enriched with the DMGs, consistent with the findings of a previous study (Wang et al., 2015a). Ca^2+^ is necessary for HIF-α translation, which is considered a key transcriptional regulator of cellular and developmental responses to hypoxia (Hui et al., 2006; Wang et al., 1995). Other research has shown an enhanced capacity of fatty acid metabolism in deer mice living at high altitudes and an expansion of genes linked to energy metabolism in the ground tit on the Tibetan plateau (Cheviron et al., 2012; Qu et al., 2013). We identified DMGs that participate in energy metabolism, particularly fatty acid metabolism, thus suggesting that Tibetan chickens adapted to hypoxic conditions enhance their energy metabolism through downregulated DNA methylation in these gene regions.

Bone morphogenetic proteins (BMPs) interact with specific bone morphogenetic protein receptors (BMPRs) on the cell surface, which are transmembrane serine/threonine kinases (Dyer, Pi & Patterson, 2014). Both BMPR1B and BMPRII belong to BMPRs, which are involved in BPM signaling pathways and hypoxia responses (De Vinuesa et al., 2016). BMPRII mediates BMP9-downregulated apelin expression in microvascular endothelial cells exposed to hypoxia, which blocks apelin-induced endothelial proliferation and promotes hypoxia-induced angiogenesis (Poirier et al., 2012). The loss of BMPRII in endothelial cells promotes endothelial apoptosis (Long et al., 2015; Teichert-Kuliszewska et al., 2006). On the basis of the DMR results, we found that BMPRII was down-methylated in TC compared to CH. We infer that this promotes its expression and further influences endothelial apoptosis. In addition, the BMP signaling pathway can exhibit various degrees of crosstalk with other pathways essential to vascular development, such as the VEGF and Wnt signaling pathways (Morrell et al., 2016; Shao et al., 2009). Although the BMP signaling pathway was not enriched in KEGG pathway analysis in this study, the VEGF signaling pathway and Wnt signaling pathways were detected.

In infarcted nude rats, HIF-1α induces the expression of *ITGA2* and consequently improves cardiac function, angiogenesis, and cardiomyocyte proliferation (Cerrada et al., 2013). Analysis using genome sequencing and ChIP has shown that the *ITGA2* gene assists hypoxic adaptation in Tibetan pigs and great tits in the highlands (Ai et al., 2014; Qu et al., 2015). In the present study, *ITGA2* was down-methylated in promoter regions, resulting in the upregulation of gene expression in TC compared to CH. We propose that the high *ITGA2* expression regulated by its low methylation level in TC embryos improves angiogenesis and cardiac function for hypoxic adaptation.

## Conclusions

In summary, we obtained the DNA methylation profiles of heart tissue from TC embryos, as well as from embryos of a related lowland breed (CH) under hypoxic conditions. Through genome-wide scanning of reads, 103,293 and 107,920 peaks were obtained in TC and CH embryos, respectively, and 1,049 DMRs (and 695 related DMGs) were screened, which are mainly involved in vascular smooth muscle contraction, VEGF signaling pathway, calcium signaling pathway, TGF-beta signaling pathway, and fatty acid metabolism. The genes *EDNRA*, *EDNRB2*, *BMPR1B*, *BMPRII*, and *ITGA2* might play roles in hypoxic adaptation through DNA methylation in TC embryos. However, further studies to validate the MeDIP-seq data using bisulfite PCR are needed. Our research provides new insights into the epigenomic regulation necessary for adaptation to hypoxia in highland animals.

##  Supplemental Information

10.7717/peerj.3891/supp-1 Figure S1 Distribution of MeDIP-seq reads on each chromosome in the Tibetan chicken (TC) and the Chahua chicken (CH) The *x*-axis indicates the number of windows, and the *y*-axis indicates the normalized read count of each window (10 kb).

10.7717/peerj.3891/supp-2 Figure S2 Distribution of the MeDIP-seq reads in the 1 kb windows with various CpG densities The *x*-axis indicates the range of CpGs in 1,000 bp, and the *y*-axis indicates the proportion of reads in a specific range.

10.7717/peerj.3891/supp-3 Figure S3 Distribution of reads in different genome elements The *x*-axis indicates different genome elements, and the *y*-axis indicates the proportion of reads in a specific gene element.

10.7717/peerj.3891/supp-4 Figure S4 The expression of five genes validated by qRT-PCR in heart tissue of chicken embryos The *y*-axis represents the expression value of mRNA in heart tissue of chicken embryos, and the *x*-axis represents names of five mRNAs. Each bar represents the mean ± S.E. for each group with 5–7 samples. Letters indicate significant differences (*p* < 0.05).

10.7717/peerj.3891/supp-5 Table S1 The primers of five DMGs and HPRT as a reference gene for qRT-PCR

10.7717/peerj.3891/supp-6 Table S2 A summary of the MeDIP-seq data

10.7717/peerj.3891/supp-7 Table S3 The genome mapping of the MeDIP-seq reads

10.7717/peerj.3891/supp-8 Table S4 A statistic of peaks on genome of the Tibetan chicken (TC) and the Chahua chicken (CH)

10.7717/peerj.3891/supp-9 Table S5 The list of the 1049 DMRs and related 695 DMGs

10.7717/peerj.3891/supp-10 Table S6 Gene Ontology (GO) functional analysis of differentially methylated genes (DMGs)

10.7717/peerj.3891/supp-11 Table S7 The KEGG pathways significantly enriched by the 695 DMGs

## Additional Information and Declarations

### Data Availability

The following information was supplied regarding data availability: GEO: GSE99129.
