# Supplementary figures and images for: Genome methylation and regulatory functions for hypoxic adaptation in Tibetan chicken embryos
Source: PeerJ. 2017 Oct 6;5:e3891. doi: 10.7717/peerj.3891 (PMC5633026; doi:10.7717/peerj.3891)

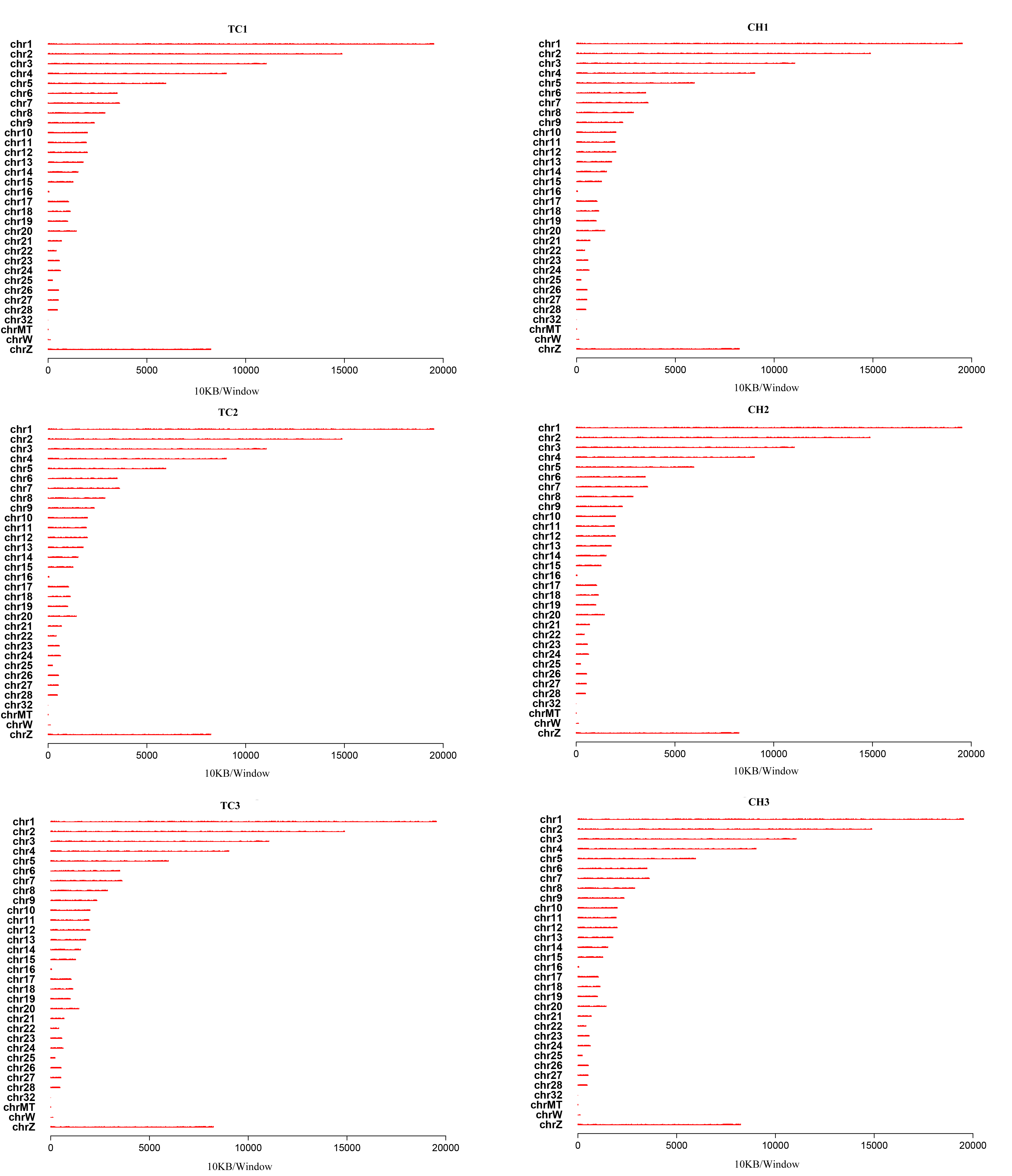

Supplement: Figure S1 — The x-axis indicates the number of windows, and the y-axis indicates the normalized read count of each window (10 kb). [file peerj-05-3891-s001.jpg]

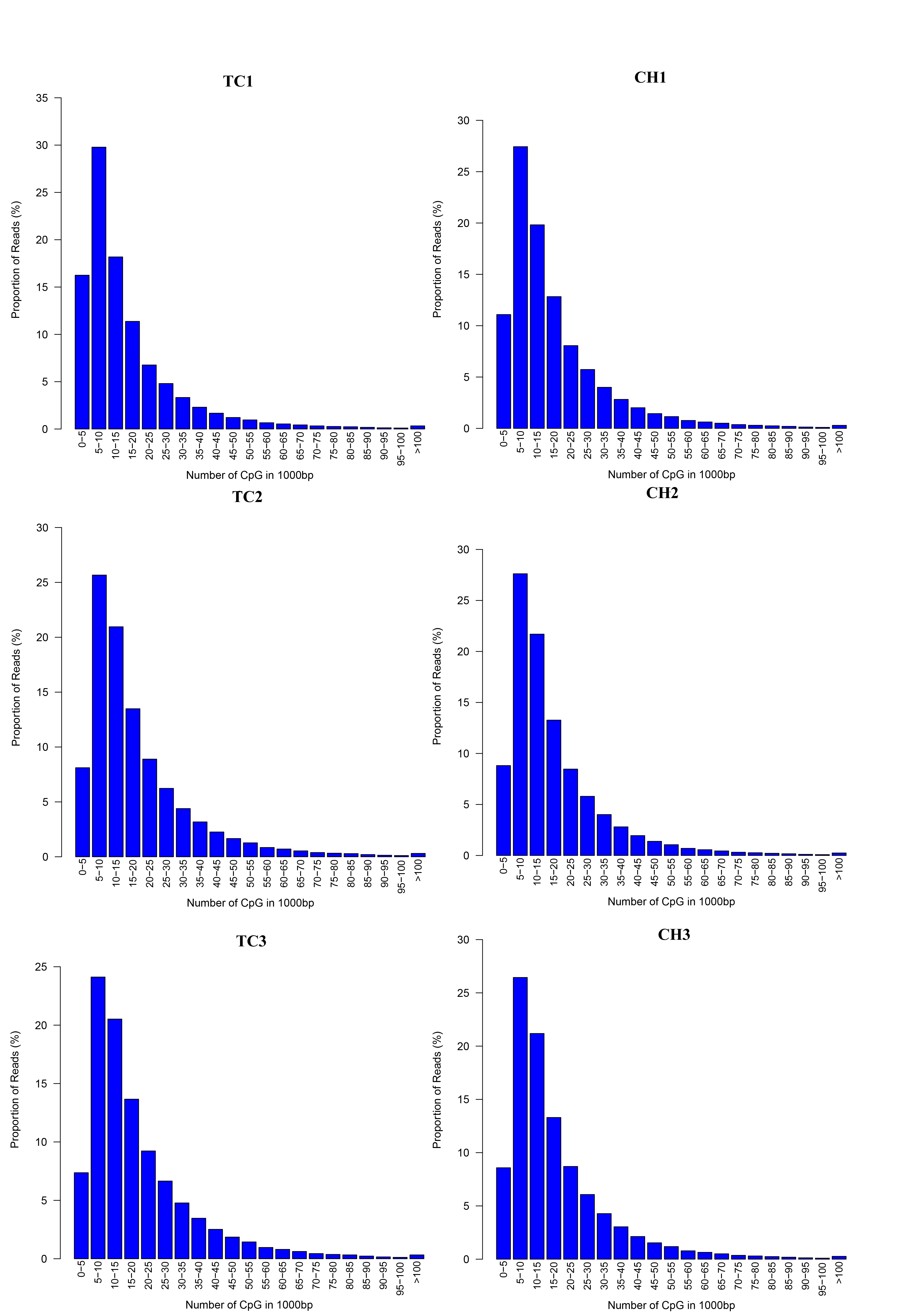

Supplement: Figure S2 — The x-axis indicates the range of CpGs in 1,000 bp, and the y-axis indicates the proportion of reads in a specific range. [file peerj-05-3891-s002.jpg]

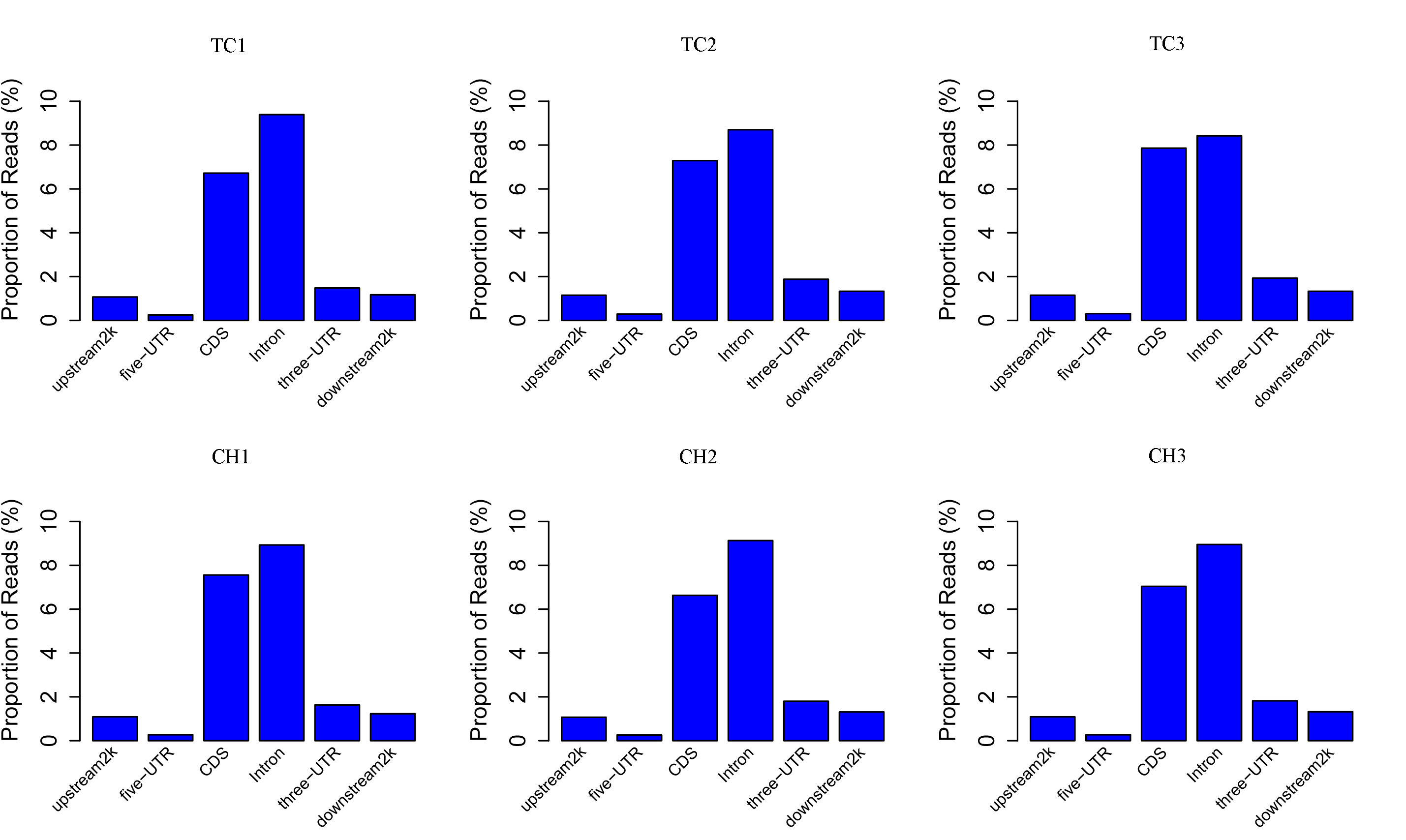

Supplement: Figure S3 — The x-axis indicates different genome elements, and the y-axis indicates the proportion of reads in a specific gene element. [file peerj-05-3891-s003.jpg]

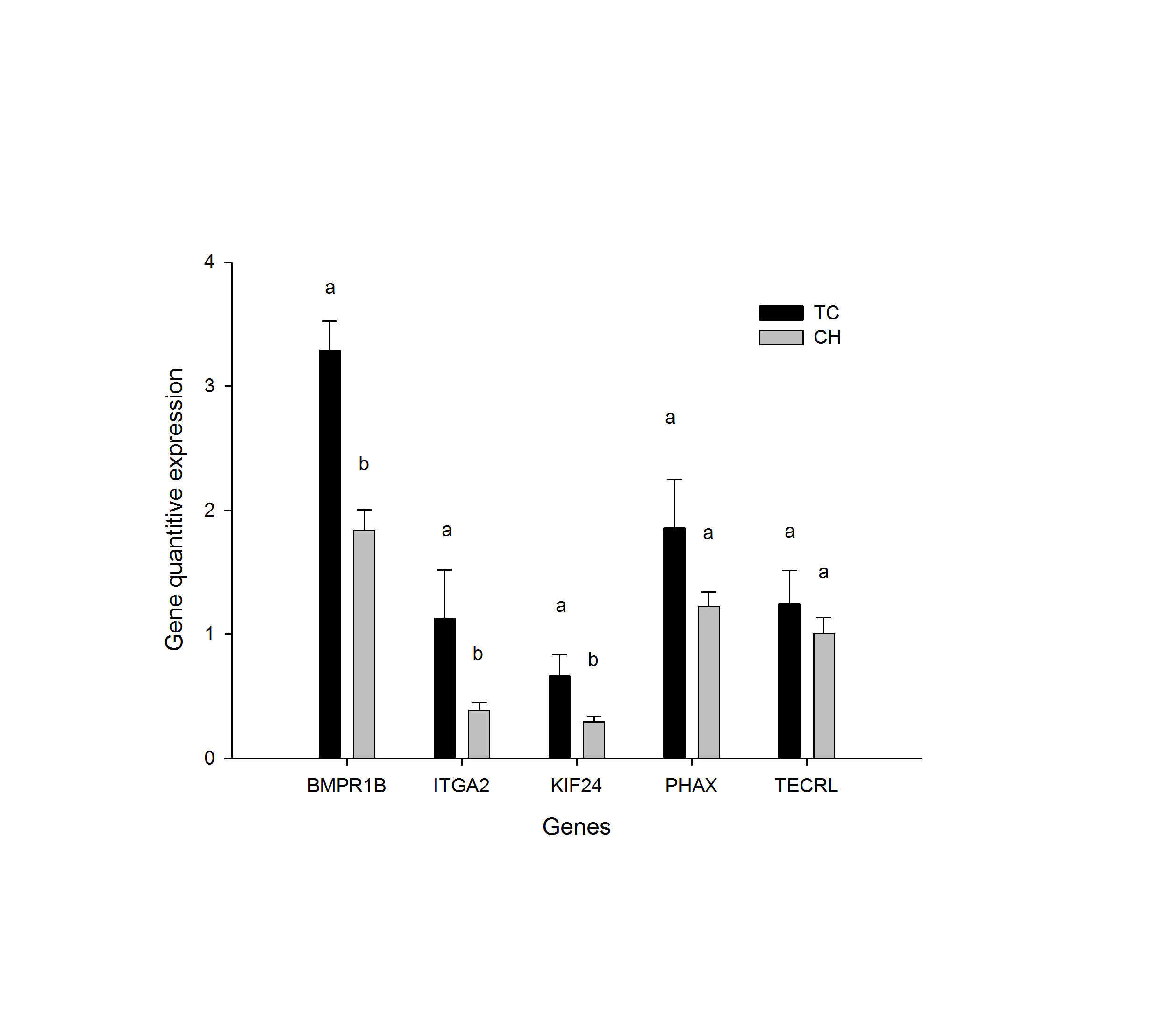

Supplement: Figure S4 — The y-axis represents the expression value of mRNA in heart tissue of chicken embryos, and the x-axis represents names of five mRNAs. Each bar represents the mean ± S.E. for each group with 5–7 samples. Letters indicate significant differences (p < 0.05). [file peerj-05-3891-s004.jpg]
